# Paraplegia after Hypotension with Pneumothorax Episode during Thoracic Spine Tumor Surgery

**DOI:** 10.1155/2021/2232769

**Published:** 2021-10-23

**Authors:** Zhao-Quan Liu, Jen-Hsien Liao, Chih-Ju Chang

**Affiliations:** ^1^Department of Neurosurgery, Cathay General Hospital, Taipei City, Taiwan; ^2^Department of Anesthesiology, Cathay General Hospital, Taipei City, Taiwan; ^3^Department of Medicine, School of Medicine, Fu Jen Catholic University, New Taipei City, Taiwan; ^4^Department of Mechanical Engineering, National Central University, Taoyuan City, Taiwan

## Abstract

Paraplegia after spine surgery is a catastrophic complication. Here, we present a patient who, following laminectomy and fusion for decompression of metastatic tumor, developed paraplegia. We tried to find out the possible reason for the paraplegia. Due to prolonged hypotension during operation and new onset of pneumothorax, we think that intraoperative prolonged hypotension leads to the spinal cord ischemia which may cause neurological deterioration of paraplegia. Maintaining hemodynamic stability during spinal surgery is very important.

## 1. Introduction

Paraplegia after spine surgery is a severe complication. Hypotension (highly suspected to be caused by pneumothorax)-induced paraplegia is an uncommon situation. Here, we present a patient who, following laminectomy and fusion for decompression of metastatic tumor, developed paraplegia. We tried to find out the possible reasons for the paraplegia immediately after the operation and discovered that the patient had new-onset pneumothorax. Be that as it may, prolonged hypotension had caused ischemia condition for the spinal cord which might induce paraplegia.

## 2. Case History

**Figure 1:**
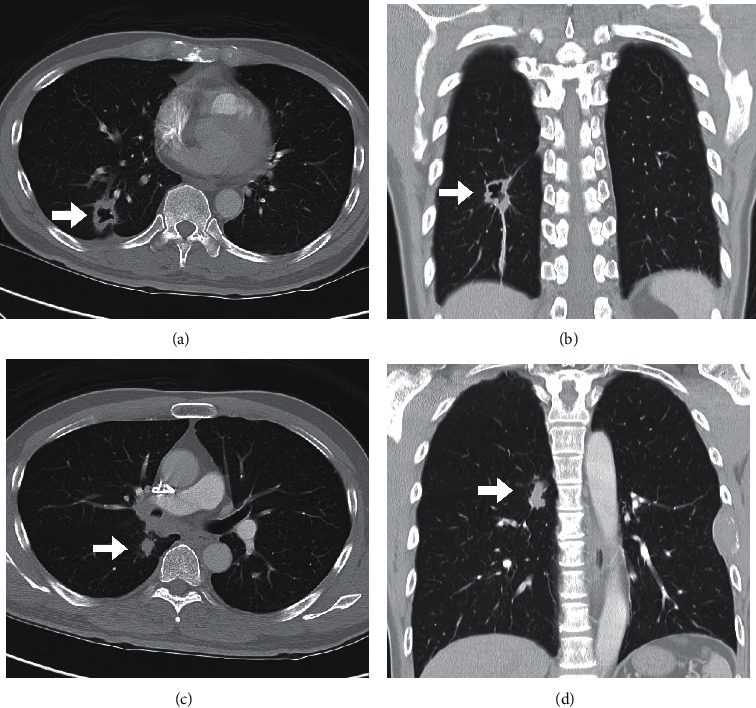
Low-dose lung CT demonstrated multiple right lung tumors (arrow).

**Figure 2:**
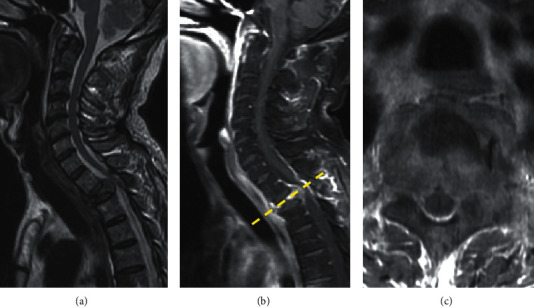
The patient presented spinal tumor at the T2 level with cord compression. (a) T2-weighted, (b) T1-weighted with a contrast sagittal view, and (c) T1-weighted with a contrast axial view.

The patient is a 62-year-old male who has right lung cancer with multiple metastasis, cT4N2M1c, stage 4B (Figure 1), after radiotherapy and chemotherapy. During the treatment protocol period, he complained about severe neck pain, bilateral arm numbness, and radiation to the subscapular area. He also had left upper limb muscle weakness. His lower limb had normal muscle power and no urine incontinence problems. The following T-spine MRI showed pathologic fracture at the T2 vertebral body (Figure 2). The patient's symptoms were related to pathologic fracture which was caused by the T2 vertebral body and T2 left-side pedicle tumor invasion. Therefore, radiculopathy and bone pain were chief symptoms instead of myelopathy.

**Figure 3:**
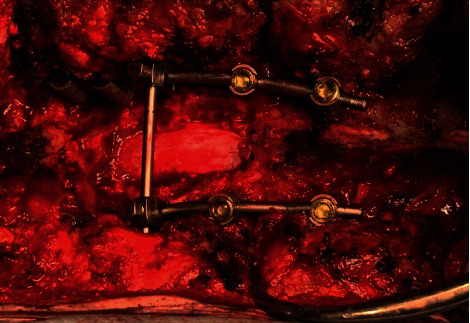
Intraoperative picture revealed the good decompression of the spinal cord.

**Figure 4:**
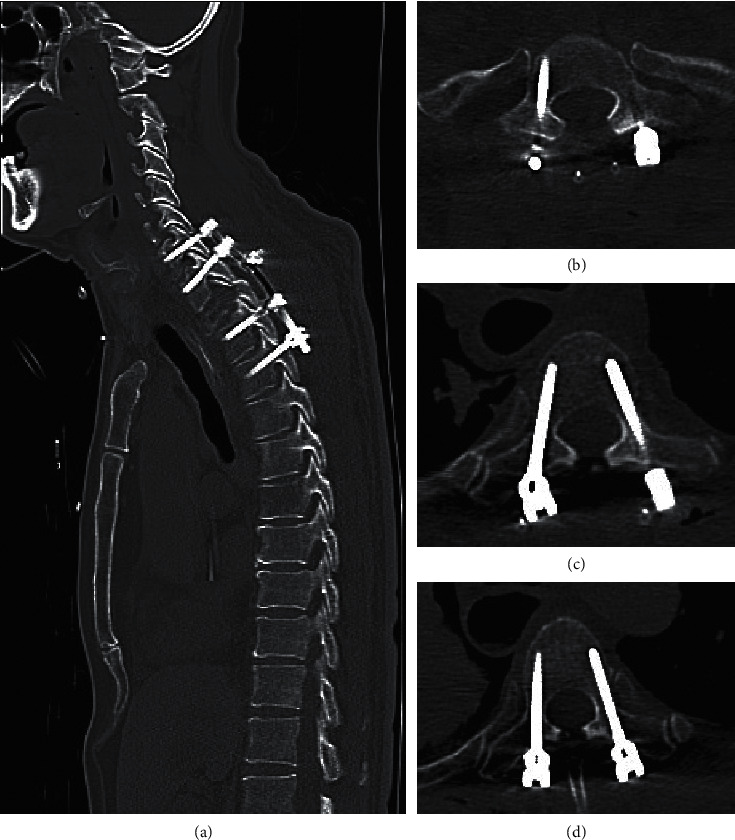
Postop immediate CT revealed all screws had good position: (a) sagittal view, (b) T1 level screws, (c) T3 screws, and (d) T4 screws.

**Figure 5:**
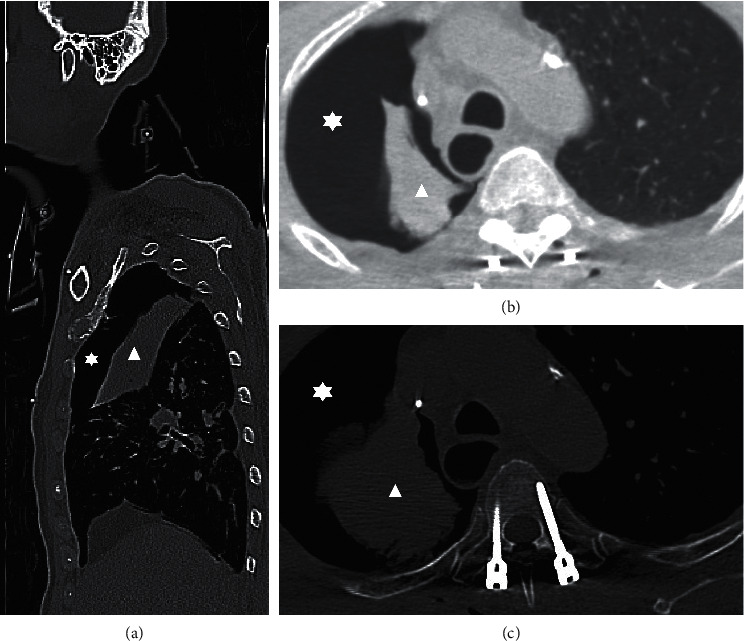
Postop immediate CT showed right apical lung pneumothorax (star) and consolidation at the right lung, leading to the radiologist's suspicion of tumor or collapsed lung (triangle): (a) sagittal view; (b) lung window.

The patient received adequate laminectomy and fusion surgery on purpose of decompression and stability of thoracic spine in order to relieve symptoms. During the operation, the patients' vital signs were stable, there was no excess blood loss, and the intraoperation somatosensory evoked potential (SSEP) revealed no special changes. Unfortunately, after 2 hours of surgery, just as we had removed the laminae to get well decompression (Figure 3), put all of pedicle screws, and prepared to fix the pedicle screws, sudden-onset blood pressure drop from 110/80 to 75/50 occurred and heart rate elevated to 120 bpm. Large amount of fluid challenge (2200 ml, including colloid and crystalloid fluid) and inotropic agent of norepinephrine (4 mg in 500 cc 5% glucose run 20 ml/hr) were given until the end of operation. According to the anesthesia record sheets, the patient's saturation was around 95∼99% during the shock condition. We completed the surgery as soon as possible. However, the shock status persisted for 1.5 hours in total even after surgery was completed. The estimated blood loss was about 250 ml, and total urine output was about 500 ml. The postoperation physical examination revealed muscle power score 0 at bilateral lower limbs. MRI would have been the best tool for assessing the epidural space and spinal cord condition. In our urgent condition, an immediate CT scan was performed instead to evaluate the condition, which showed all pedicle screws well positioned (Figure 4). The radiologist opined that there was no obvious compressive lesion around the spinal cord such as a hematoma or abscess between C1 and T9 but found right upper lung pneumothorax with obvious bullae (Figure 5). We then performed the needle thoracotomy at once; the following chest X-ray showed persistence of pneumothorax nevertheless.

When we wanted to arrange the further MRI examination, the patient's family hesitated and denied to receive the further examination or other aggressive treatment. The patient was managed with lower extremity physiotherapy and conservative treatment for terminal cancer; after 2 weeks of admission follow-up, however, no evidence of lower limbs' recovery was detected and the patient chose hospice management.

## 3. Discussion

Paraplegia is a catastrophic complication in spine surgery. There are various possible causes for paraplegia. However, the risk cannot be precisely quantified because of the lack of current data. In Delank et al.'s study [1], out of the 1194 patients surveyed, 7 (0.59%) experienced a postsurgical complete or incomplete paraplegia in scoliosis thoracic spine surgery [2].

Some studies report [3] that paraplegia may be caused by Adamkiewicz artery injury at the T3 to T6 level. Another possible cause of paraplegia was “white cord syndrome”(WCS) [4] which means sudden neurological deterioration following spinal decompression may be attributed to a reperfusion injury of the spinal cord. The spine cord edema extends to the cervical level or even the brain stem. Iatrogenic epidural hematoma-caused paraplegia also had been reported [5] as immediate postoperative period neurological deterioration. Furthermore, cord injury-caused paraplegia due to the pedicle screws malposition was also noted, especially at scoliosis patients [6].

Although the etiology of sudden-onset paraplegia remains obscure, the most important thing to prevent neurological deterioration such as paraplegia after posterior decompression surgery is to maintain the mean arterial pressure (MAP) during the spine surgery [7]. According to Kobrine et al.'s experimental study, spinal cord blood flow (SCBF) had autoregulation between MAP 50 to 135 mmHg. However, below 50 mmHg, further decreases in MAP effected corresponding decreases in the SCBF. The vessels were maximally dilated, vascular resistance was maximally reduced, and SCBF was a function of MAP [8].

In this respect, consensus [9] of preoperative optimization of volume status and aggressive maintenance of MAPs more than 110% of preoperative values in the procedure of spinal cord decompression were recommended in the surgical management of thoracic spine surgery.

The recommendation mentioned above contradicts the proper intraoperative MAP, as low blood pressure leads to spinal cord ischemia, whereas high pressure may cause excessive bleeding which could decrease the venous return [10] Therefore, the effective measures to prevent cord ischemia are to improve the surgical technique, shorten operation time, and pay more attention to hemostasis.

In hindsight, the blood supply of the thoracic spinal cord is much less collateralized than the cervical and lumbosacral regions, resulting in a greater risk of ischemia and infarction in conditions where blood flow is compromised [11]. The blood flow (I) is related to perfusion pressure (V) and resistance (R) (I = V/R). As tissue interstitial pressures secondary to pressure from the thoracic cavity (such as pneumothorax), spinal cord perfusion pressures decline. In an attempt to help mitigate the drop in blood flow, autoregulatory mechanisms result in a decrease in vascular resistance at the level of the penetrating arteriole.

This case includes clinical and radiological features related to hypotension-induced spinal cord ischemia change and paraplegia. We proposed the hypothesis that intraoperative hypotension might induce paraplegia and the pneumothorax could cause hypotension.

The possible cause of pneumothorax during the procedure was tumor invaded to the pleura or pulmonary space. During the surgery by the posterior approach for decompression, the tension was released and pneumothorax was caused. The other possible reason may be primary lung lesion and prone position; both of them increased the aveolar positive end-expiratory pressure (PEEP) and induced pneumothorax. Intraoperative pneumothorax is a rare situation, but sometimes, it can potentially be life threatening [12]. The presence of emphysematous bullae or smaller blebs is a situation in which pneumothorax develops with positive pressure ventilation due to bullae or blebs rupture. On the other hand, an increase in central venous pressure can result in various situations such as distended neck veins and hypotension. Patients' clinical condition may have tachypnea, dyspnea, tachycardia, and hypoxia.

Moreover, some other reasons could cause unstable intraoperative hemodynamics including the impairment of spinal venous drainage, arterial thrombosis, inflammatory processes, or secondary injury processes. Inadvertent cord handling rarely causes the extension of edema more than two levels above the injured segment and should be evident in the immediate postoperative period.

Most of procedures were normal during the entire operation, except the unexplained shock condition. Also, after the operation, a newly occurring pneumothorax was found. We inferred that the patient's paraplegia is caused by shock which maybe induced by pneumothorax. In order to minimize the risk of paraplegia after thoracic spinal decompression, we strongly recommend that maintenance of hemodynamic stability and the carefulness about any possible reasons which may cause spinal cord ischemia. When the patient had hypotension status, pneumothorax may be the one of the possible reasons. Clinicians should be very careful of this devastating complication and include it during the informed consent-taking process preoperatively.

## Data Availability

The data are available on request from the authors.

## References

[1] Delank K. S., Delank H. W., Konig D. P., Popken F., Furderer S., Eysel P. (2005). Iatrogenic paraplegia in spinal surgery. *Archives of Orthopaedic and Trauma Surgery*.

[2] Noonan K. J., Walker T., Feinberg J. R., Nagel M., Didelot W., Lindseth R. (2002). Factors related to false- versus true-positive neuromonitoring changes in adolescent idiopathic scoliosis surgery. *Spine (Phila Pa 1976)*.

[3] Laratta J. L., Shillingford J. N., Saifi C., Riew K. D. (2018). Cervical disc arthroplasty: a comprehensive review of single-level, multilevel, and hybrid procedures. *Global Spine Journal*.

[4] Vinodh V., Rajapathy S., Sellamuthu P., Kandasamy R. (2018). White cord syndrome: a devastating complication of spinal decompression surgery. *Surgical Neurology International*.

[5] Jusué-Torres I., Ortega-Zufiria J. M., Tamarit-Degenhardt M. (2011). Hematoma epidural cervical yatrogénico. Presentación de un caso clínico y revisión de la literatura. *Neurocirugía*.

[6] Mac-Thiong J. M., Parent S., Poitras B., Joncas J., Hubert L. (2013). Neurological outcome and management of pedicle screws misplaced totally within the spinal canal. *Spine (Phila Pa 1976)*.

[7] Marcus M. L., Heistad D. D., Ehrhardt J. C., Abboud F. M. (1977). Regulation of total and regional spinal cord blood flow. *Circulation Research*.

[8] Kobrine A. I., Doyle T. F., Rizzoli H. V. (1976). Spinal cord blood flow as affected by changes in systemic arterial blood pressure. *Journal of Neurosurgery*.

[9] Zuckerman S. L., Forbes J. A., Mistry A. M. (2014). Electrophysiologic deterioration in surgery for thoracic disc herniation: impact of mean arterial pressures on surgical outcome. *European Spine Journal*.

[10] Wang H., Ma L., Xue R. (2016). The incidence and risk factors of postoperative neurological deterioration after posterior decompression with or without instrumented fusion for thoracic myelopathy. *Medicine*.

[11] Martirosyan N. L., Feuerstein J. S., Theodore N., Cavalcanti D. D., Spetzler R. F., Preul M. C. (2011). Blood supply and vascular reactivity of the spinal cord under normal and pathological conditions. *Journal of Neurosurgery: Spine*.

[12] Heyba M., Rashad A., Al-Fadhli A. A. (2020). Detection and management of intraoperative pneumothorax during laparoscopic cholecystectomy. *Case Reports in Anesthesiology*.

